# The Application of Massage (*Kneading*) and Steam, in Abscess of the Cornea

**Published:** 1874-12-15

**Authors:** 


### THE APPLICATION OF MASSAGE (*KNEADING*) AND STEAM, IN ABSCESS OF THE CORNEA.

*Translated from La France Medicale, by Fred. J. Huse, M.D.*

In a recent number of the *Independencia Medica*, of Barcelona, Dr. Osio deplores the inefficiency of our therapeutics in the treatment of a large number of maladies, and he maintains that this seeming lack of means for successful medication has its origin in the fact that many very simple and highly efficacious remedies are entirely disdained by the physician, and remain among the number of those medicines commonly called old woman's remedies.

Mackenzie mentions an alarming epidemic of ophthalmia, which broke out among the cargo and crew of the slave-ship Rodeur, and cites the relief that was immediately afforded, upon arriving at Guadaloupe, by the topical application of lemon juice, at the suggestion of an old negro midwife. In reading this account one is at first impressed with incredulity, but regarding it more carefully, and interpreting this circumstance by the aid of well known facts, there may be found a rational explanation. Indeed, Dr. Onimus has presented a paper to the Academy of Medicine, in Paris, in which he enumerates the substances which most surely neutralize septicæmia. The most energetic of these neutralizing agents is sulphuric acid, which, even in very minute doses, destroys the virulence of depraved organic liquids. The vegetable acids possess, in a certain proportion, the antiseptic properties of the mineral acids, and hence the efficiency of the citric acid against the purulent ophthalmia may thus be readily understood.

Massage of the cornea, which every one unconsciously practices in the act of rubbing the eyes, is one of those simple, common remedies, which can be made to render valuable service. At the Ophthalmological Congress, held in London, in 1872, the celebrated Donders called attention to a practice which might, he said, appear whimsical and valueless, but which had yielded him excellent results. He advocated massage of the cornea. Subsequently Dr. Osio practiced massage of the cornea in certain ocular diseases, and invariably with most fortunate results. More recently he has combined the use of vapor of hot water with massage.

It is well known that in addition to ocular abscesses of traumatic origin there exist others of pathological origin, and of inflammatoiy nature, others of diasthetic nature, and still others which have their origin in the surroundings in which the subjects live. Those ocular suppurations which depend upon lymphatic diathesis, scrofula, moist climate, etc., produce a lesion that is entirely asthenic. Slowly, without pain, without delacrymation, and almost without injection of the tissues of the eye, pus forms, penetrates the layers of the cornea, distends and ruptures its elastic posterior membrane, forms an enormous hypopyon, and partially fills the anterior chamber. It is in cases such as these that massage and steam produce most prompt and happy results.

Dr. Osio employs the following method: An apparatus charged with an infusion of camomile, is placed before the patient's eyes, which have previously been covered with a double layer of fine muslin. The apparatus should be placed at a sufficient distance that the vapor may reach the eyes at a temperature of from 90° to 100°. At the same time massage of the eye should be performed with the fingers over the muslin, rubbing it up and down, from side to side, and finally by a circular movement pressing upon the centre of the cornea.

At intervals the apparatus may be brought nearer the patient so that the eyes may for a few moments be subjected to steam of a higher temperature than that already indicated.

This vapor bath should be continued for a half or three-quarters of an hour, and during this time the massage should be repeated from eight to ten times, with a duration of from one to two minutes upon each occasion. At the termination of the sitting a drop of a collyrium containing atropine should be used, and last of all a retentive bandage.

The general treatment should be metasyncritic and the diet generous, while to subjects seriously tainted with scrofula should be administered Van Swieten's liquor (10 grains of corrosive sublimate to 2½ pounds of brandy).

The beneficial action of this method compares favorably with those obtained by Mackenzie and Weeker, with compresses of warm water in preventing suppuration of the cornea. It may be resorted to in asthenic abscesses, as well as in ocular inflammations, for the purpose of preventing attachment or prolapse of the iris.

